# Metabolomic Laboratory-Developed Tests: Current Status and Perspectives

**DOI:** 10.3390/metabo11070423

**Published:** 2021-06-26

**Authors:** Steven Lichtenberg, Oxana P. Trifonova, Dmitry L. Maslov, Elena E. Balashova, Petr G. Lokhov

**Affiliations:** 1 Metabometrics, Inc., 651 N Broad St, Suite 205 #1370, Middletown, DE 19709, USA; 2 Institute of Biomedical Chemistry, 10 Building 8, Pogodinskaya Street, 119121 Moscow, Russia; oxana.trifonova@gmail.com (O.P.T.); dlmaslov@mail.ru (D.L.M.); balashlen@mail.ru (E.E.B.)

**Keywords:** diagnostics, laboratory-developed test, in vitro diagnostic device, metabolomics, mass spectrometry, dried blood spot, metabolomic study

## Abstract

Laboratory-developed tests (LDTs) are a subset of in vitro diagnostic devices, which the US Food and Drug Administration defines as “tests that are manufactured by and used within a single laboratory”. The review describes the emergence and history of LDTs. The current state and development prospects of LDTs based on metabolomics are analyzed. By comparing LDTs with the scientific metabolomics study of human bio samples, the characteristic features of metabolomic LDT are shown, revealing its essence, strengths, and limitations. The possibilities for further developments and scaling of metabolomic LDTs and their potential significance for healthcare are discussed. The legal aspects of LDT regulation in the United States, European Union, and Singapore, demonstrating different approaches to this issue, are also provided. Based on the data presented in the review, recommendations were made on the feasibility and ways of further introducing metabolomic LDTs into practice.

## 1. Introduction

Metabolomics is the comprehensive study of the metabolome as a set of all substances present in biological samples with a molecular weight of <1000 Da [1]. The metabolome mainly consists of metabolites that are substrates, the end products of biochemical reactions that take place in cells. At the center of metabolomics is the concept that the metabolite composition of bodily fluids may accurately describe the physiological and pathological state of the organism. In particular, metabolomic studies of blood samples made it possible to diagnose many human diseases with a diagnostic accuracy of 90–95% [2]. It has also been shown that the metabolome of urine, saliva, and cerebrospinal fluid can be effectively used for disease diagnostics [3,4,5].

It is also worth noting the opinion of the Metabolomics Society, which is that the study of the organism at the global or “-omics” level is a rapidly growing field that can have a profound impact on medical practice. Today, doctors use only a very small part of the information contained in the metabolome, as they usually measure only a narrow subset of substances in bodily fluids to assess health and disease. It is expected that “the narrow range of chemical analyses in current use by the medical community today will be replaced by analyses that reveal a far more comprehensive metabolic signature in the future. This signature is expected to describe global biochemical aberrations that reflect patterns of variance in states of wellness, more accurately describe specific diseases and their progression, and greatly aid in the differential diagnosis” [6].

Today researchers try to create omics tests to diagnose diseases, assess the risk of their development, and determine the patient’s response to treatment [7]. However, despite the prospect of introducing omics tests into clinics, their application in practice is rather difficult due to the lack of regulated procedures and standards, the development of which is difficult due to their complexity [8].

A possible solution to this problem seems to be the introduction of metabolomics into clinical practice in the laboratory-developed test (LDT) format, which has been widespread in medical practice for decades. The LDT is a subset of in vitro diagnostic devices (IVDs), which is designed, manufactured, and used within a single laboratory [9,10,11,12,13]. 

LDT can be used to measure a wide variety of analytes in human samples. There are fairly simple LDTs that measure individual analytes. However, there are also more sophisticated and complex LDTs, such as omics tests, with which it is possible to measure a large number of analytes. Several LDTs have been published for the diagnosis of various diseases, including genetic disorders, cancer, infections, and other disorders [14,15,16,17,18,19,20]. Among them, research papers on metabolomics have been published that describe the early diagnosis of Parkinson’s disease [21,22].

The purpose of this publication is to review the existing metabolomic LDTs, assess the prospects for the development and scaling of metabolomic LDTs, and the potential for their use in clinical medicine. Due to some similarities between clinical laboratory tests, laboratory methods of scientific research, and LDT workflow, for a clearer understanding of metabolomic LDT, they were all compared with each other. In addition, an overview of LDT regulations in the United States, European Union, and Singapore is provided.

## 2. History of LDT

IVDs are “those reagents, instruments, and systems intended for use in the diagnosis of a disease or other conditions, including a determination of the state of health, in order to cure, mitigate, treat, or prevent disease or its sequelae. Such products are intended for use in the collection, preparation, and examination of specimens taken from the human body” [9]. LDT is a subset of IVDs, which the US Food and Drug Administration (FDA) defines as “in vitro diagnostic tests that are manufactured by and used within a single laboratory”, i.e., a laboratory with a single Clinical Laboratory Improvement Amendments (CLIA) certificate. LDTs are also referred to as in-house developed tests or “homebrew” tests [23]. 

The history of the appearance and regulation of LDTs begins in 1976, when, as part of the Medical Device Amendments to the Federal Food, Drug, and Cosmetic Act (FD&C Act), Congress gave the FDA the authority to regulate IVDs as medical devices [24]. This was done in order to create a regulatory framework that includes the risk-based classification system for medical devices and the premarket review process. While the FDA has developed a robust regulatory process for IVDs premarket validation, it has adopted a decidedly hands-off approach to regulating LDTs. The FDA has assigned LDTs a low risk due to their limited availability and their primary use in the context of rare diseases. Therefore, LDTs have not undergone rigorous premarket evaluation. The result is a bifurcated market in which IVDs designed for commercial sale meet stringent FDA standards, while LDTs designed for the same purpose within a single laboratory do not.

Over the past 40 years, medical and technological advances have spurred an increase in LDTs to diagnose a wide range of diseases, including human papillomavirus (HPV) [25], Lyme disease [26], whooping cough [27], some cancers [15,17], and heart disease [28,29]. The growth of the LDTs segment has raised concerns about whether the current regulatory oversight of LDTs, primarily by the Centers for Medicare and Medicaid Services (CMS), is sufficient to ensure its safety and effectiveness. Reports of inaccuracies in cervical cancer LDTs led to the CLIA of 1988, which extended federal rules to all laboratories testing human samples for diagnosis or treatment. The rules that have been in effect since 1994 have remained largely unchanged since then. LDT is currently regulated by CMS and its prospective accrediting bodies under the CLIA, as well as the New York State Department of Health, which has its own LDT regulations. 

In July 2010, the FDA announced its intention to review its LDT regulatory policy. In 2014, the FDA issued a draft guideline proposing a regulatory framework for LDT [9]. A year later, on 16 November 2015, a report on 20 case studies of potential and actual harm to patients caused by inaccurate or unreliable LDT was released, which confirmed the need to strengthen LDT control [24].

In January 2017, the FDA announced that it would not finalize the Framework for Regulatory Oversight of LDTs and invited Congress to address the issue, but in October 2018, the FDA issued guidance warning that many genetic tests on the market that claim to predict a patient’s response to certain medications have not been reviewed and may not be supported by scientific or clinical evidence [30,31]. In April 2019, the FDA issued a warning letter to the Inova Genomics lab claiming that its genetic tests, which were being offered for the same purpose, were tampered with and posed a serious public health concern because they had not been properly tested [32].

In the face of the COVID-19 pandemic, the FDA has included LDT in its policy for other IVDs designed for disease diagnostics. However, a statement by the Federal Department of Health and Human Services (HHS) dated 19 August 2020 overturned all previous FDA regulation statements on LDTs [33]. HHS stated that LDTs would not be subjected to FDA premarket review in the absence of official agency rulemaking. The COVID-19 pandemic and also the HHS decision provoked an increase in the number of new LDTs [34].

## 3. Metabolomic LDT

Today, the focus of health systems in developed countries is shifting from acute care to disease prevention and early medical intervention if a disease is discovered and there is a gradual transition from standardized clinical protocols to personalized medicine [35]. Furthermore, various digital innovative solutions are actively appearing on the healthcare market for simple and regular monitoring of people’s health, but they only allow indirectly assessing some health parameters at a superficial level [36]. Among the innovations in the field of healthcare, various technological solutions are also popular that integrate artificial intelligence algorithms into the work of medical organizations and pharmaceutical companies to achieve better results in their work [37,38,39,40,41,42]. 

Despite the active attempts of states and companies to make the healthcare sector easier and more accessible for all people, millions of people in developed countries do not receive recommended preventive medical services, and solutions that are created for simple and convenient monitoring of health status are often not able to produce a deep and accurate diagnosis of the health state [43]. To change this, government programs are being created in the United States to improve this situation [44]. Therefore, one of the most urgent tasks in the field of healthcare and personalized medicine is the development of technologies for convenient, comprehensive, and affordable diagnostics of the human health state and the detection of diseases at early stages. Metabolomics and its practical application and scaling in the format of LDT can help solve this difficult task. 

The basic omics sciences, such as genomics, transcriptomics, proteomics, or metabolomics, study biological objects at the level of the genome, proteome, transcriptome, and metabolome, respectively. Metabolomics is the last to appear among these omics sciences and the logical conclusion in the systemic study of biological objects. Studying the totality of low-molecular substances that are substrates, intermediates, and products of biochemical reactions, metabolomics describes the molecular phenotype of a biological object, which reflects the “realized” genome. Metabolomics is the most promising science in terms of clinical application in comprehensive diagnostics of human health among the omics sciences because it studies the molecular phenotype of a person [2,45,46,47,48]. By studying the end products of near-to-all biochemical reactions in the human body and the influence of the external stimuli (exogenous metabolites) from the environment on the body using metabolomic analysis, it is possible to detect any changes in the human body associated with lifestyle, nutrition, and pathological processes that begin or occur in the human body [49,50].

Modern metabolomics is characterized mainly by the use of methods based on mass spectrometry in conjunction with the bioinformatic treatment of data, which allows fast and complex analysis of metabolites with a high diagnostic accuracy of 90–95% [6,51,52,53,54]. The most common methods for analyzing metabolites are metabolomic fingerprinting and metabolomic profiling [55,56,57]. Metabolomics has already demonstrated impressive results: to date, several thousand mass spectrometry-based metabolomics studies have been published aimed at developing the diagnosis of diseases (including various forms of cancer, diabetes, vascular, neurodegenerative disorders, and many others) [58,59,60,61,62,63,64]. Their number is constantly increasing every year. From the point of integrating metabolomics into precision medicine, the individual metabotype is worth mentioning. People's subpopulations are characterized by distinct metabolic phenotypes, known as metabotypes [65]. The individual metabotype provides a detailed molecular snapshot representing wellness, aging, dietary intake, physical activity, stress, and other physiological factors associated with the human lifestyle. Precision medicine may provide an accurate diagnosis of the disease, its stage, the risk of actionable clinical complications by combining the metabotype with demographic, anamnestic, clinical, and genomic data.

Despite this, there are currently no clinically and FDA-approved complex metabolic diagnostic tests [66]. On 25 March 2021, a combined search for “metabolomics and laboratory test” in PubMed showed only two relevant publications, which are a prerequisite for the creation of the first comprehensive metabolomics test designed to diagnose early-stage Parkinson’s disease [21,22].

In addition, the only company that has made metabolomic LDT available is Metabolon, Inc. In 2018, they announced that the Meta UDx™ test is available to accelerate the diagnosis of rare and undiagnosed diseases in children and adults, which has been analytically validated under the CLIA as LDT [67]. Using advanced metabolomics technologies, Meta UDx™ detects abnormalities in major human metabolic pathways or biomarkers that cannot be measured by other means. For this, about 1000 metabolites are studied at one time. The Metabolon range of tests also includes Meta IMD™ and Meta IMD™+ (Plus) to diagnose rare genetic disorders known as hereditary metabolic disorders or congenital metabolic disorders. Like LDT, these tests have not been approved by the FDA, but can be used clinically as secondary tests and in combination with other standard clinical diagnostic tests [11].

Based on the above, it follows that metabolomics is an extremely promising science with great applied potential. At the moment, a large amount of scientific data has been accumulated, which must be gradually translated into practical medicine through the creation of new metabolomic LDTs for an accurate, simple, accessible, and comprehensive diagnosis of human health.

## 4. Metabolomic LDT vs. Metabolomics Study

On 28 March 2021, a search for the words “metabolomics” and “study” in the titles and abstracts among all publications in PubMed over the past 10 years shows that 11,000 scientific publications have been published, with 9000 of them published in the last 5 years. This once again indicates that metabolomics is an actively developing science, and a large amount of theoretical and practical data has been accumulated to date. It is imperative to channel the accumulated data in this area into applied medical research, which will have a positive impact on the development of the industry and the well-being of end-users in it [68,69]. 

Thus, it is necessary to find a solution to adapt and scale the metabolomics technologies to provide access to them for all people. As a possible solution, the LDT can be considered, which may solve some of the problems of classical metabolomic study and make it accessible to people. 

Among the main limitations of the classical metabolomic study, several of the most significant ones can be distinguished, which hinder its scalability and accessibility for people:

- There are no government regulations for typical scientific metabolomic studies, and they are conducted solely for research purposes and cannot be commercially scaled up;
- The cost of the scientific metabolomic study is prohibitive for ordinary people, and often such research is funded by public or private grants. Furthermore, the so-called metabolomic case studies are quite rare, which is closest to a personalized metabolomic study, but this is not a scalable research model [70,71];
- The scientific metabolomic study is difficult to reproduce. It is carried out to discover new phenomena and acquire new knowledge. It is based on the experience of a specific research team and reflects the experience and knowledge of only specific people;
- The timing of scientific metabolomic studies can range from several months to several years, which limits their availability for rapid diagnosis;
- The presentation of data in the scientific metabolomic study is available only to scientists in the field. Most people do not have access to data interpretation in scientific metabolomic research due to their specificity and complexity.

And these are just a few of the limitations that prevent the classical scientific metabolomic study from scaling up and becoming an everyday diagnostic tool in applied medical practice.

Furthermore, most of the problems can be solved using metabolomics in the LDT format. This approach solves the above problems and has several advantages over the typical scientific metabolomic study in order to scale up metabolomic methods and their application in clinics.

**Figure 1:**
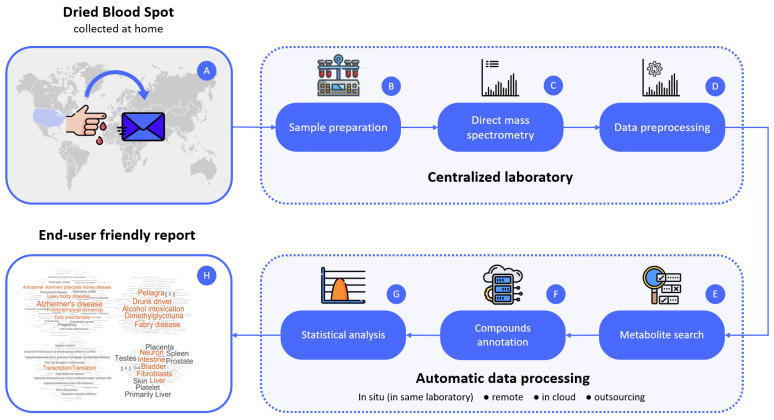
The workflow of LDT for the diagnosis of Parkinson’s disease. Blood samples (DBS) are collected (**A**) at home and transported to the laboratory via the postal service. In the laboratory, after sample preparation (**B**) and high-resolution direct mass spectrometry (**C**), the mass spectra of the blood samples are obtained. The obtained masses of compounds after preprocessing (**D**) are submitted to the metabolite search block (**E**) to find metabolite identifiers from the Kyoto Encyclopedia of Genes and Genomes database (KEGG) database matching the *m*/*z* values. Matched KEGG IDs are submitted to a compound annotation algorithm (**F**) [76], and the retrieved results are used for the overrepresented metabolite set analysis (**G**). Finally, overrepresented metabolite sets from an individual are visualized as a metabolite set names cloud, where the font size corresponds to the representation value (score) (**H**). Adapted from [22].

Among the advantages of this approach are the following:

- The LDT format simplifies the implementation of metabolomics-based tests, turning protocols and standardization actions into routines of one laboratory, which is regulated to some degree by the local rules;
- Metabolomic LDT can be implemented in the “direct to customer” format since mass spectrometric measurements in metabolomic LDT are compatible with dried blood spot (DBS) samples [72], and there are also methods for simple collection of capillary blood without assistance at home and its subsequent transportation to the laboratory by mail or using a specialized courier service, which makes LDT convenient for customers and available almost everywhere. The LDT results are user-friendly, making them acceptable to a wide range of customers.
- Any educated person can be the end-user of metabolomic LDT. LDT results are available to a wide range of clients: physicians and their patients, researchers, citizen scientists, and any educated person. The process of interpreting complex metabolomic data can be automated, and the data can be presented in an accessible format [22];
- Unlike scientific metabolomic research, LDT can be reproduced an unlimited number of times according to the created workflow (Figure 1) within one laboratory and can be used by people for independent research of the state of health of their body and regular monitoring of their health, which has a pronounced applied value in the modern world [22];
- The method of direct mass spectrometry of blood plasma, based on which it is possible to implement metabolomic LDT, is characterized by a high processing speed and relatively high reproducibility and was also widely used in metabolomics, in particular, in the laboratory where LDT was developed for the study of cancer, diabetes [73], and Parkinson’s disease [74]. The processing of mass spectrometric data was specially developed for high-resolution mass spectra and was successfully used for many years in studies of blood plasma [75], and now it is implemented in the LDT format [22];
- In the LDT format, various options for diagnosing human health can be implemented, for example, as “confirmation of a person’s healthy state”, “score-based diagnostics”, and “disease diagnosis based on metabolite set overrepresentation” [22]. In the future, it is possible to create new and more adaptive methods for diagnosing the state of human health;
- Due to the potentially large number of tests within one laboratory, the cost of metabolomic LDT is expected to be quite low and acceptable for most people.

Moreover, at the moment, a metabolomic LDT workflow has been developed (Figure 1), which describes a model step-by-step for conducting a metabolic study of blood plasma samples or human capillary blood in the form of a dried drop. This process allows metabolic studies to be standardized and scaled, making them accessible and convenient for most people.

**Figure 2:**
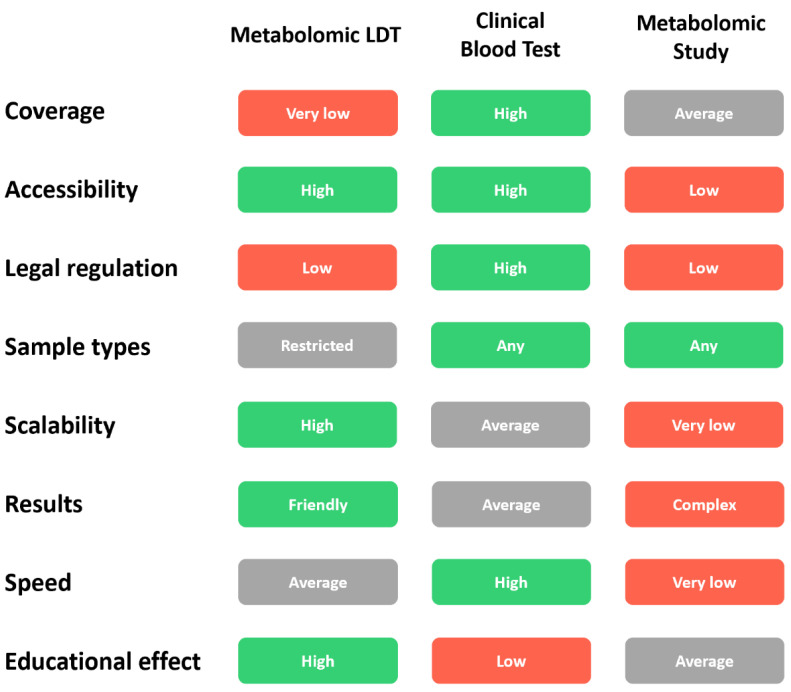
Comparative analysis of metabolomic LDT vs. clinical blood test and scientific metabolomic study.

A general comparison of metabolomic LDT with existing clinical blood tests and scientific metabolomic studies was also made to assess the prospects for scaling up and using metabolomic LTD in clinical practice (Figure 2). 

Comments on the comparison of metabolomic LDT with a typical clinical blood test and metabolomic study in science for several parameters are presented in Table 1.

Based on the above, it can be concluded that there is an opportunity to develop a personalized metabolomic LDT for comprehensive diagnostics of human health. Metabolomic LDT meets four key criteria for a viable market model: affordable price, availability of test results to the end-user, fast testing speed, and scalability. In addition, metabolomic LDT is potentially more convenient for end-users, multifunctional, and more informative than clinical blood diagnostics.

Although only blood was used for comparison in Table 1, as in this case the most representative biological fluid, the main points of comparison are most likely true for urine. The analysis of urine is undoubtedly also important from the point of the development of LDT.

## 5. Analytical Limitations 

Although mass spectrometry-based metabolomics has many advantages and prospects, and its representation in the form of LDT can be a way of scaling metabolomics technologies for comprehensive diagnostics of human health, nevertheless, there are several limitations. 

Speaking about the limitations of LDTs, one cannot fail to mention the FDA document “The Public Health Evidence for FDA Oversight of Laboratory Developed Tests: 20 Case Studies”, which was created by the FDA in 2015 to highlight the shortcomings of the current LDT’s regulation system, which leads to the emergence of false-positive and incorrect data as a result of some studies, as well as the lack of an exhaustive list of existing LDTs, makes it difficult to understand the real picture in this segment [24,77].

Some of the limitations and tasks that faced metabolomics 10 years ago [78] have now been solved, but there are also many tasks to be solved [48,58,79,80]. Mass spectrometers, nuclear magnetic resonance (NMR) spectrometers, and other equipment that are required to conduct the metabolomic study are expensive and require experienced staff to operate this equipment. Along with this, there are several different methods of mass spectrometry: gas chromatography-mass spectrometry (GC-MS), liquid chromatography-mass spectrometry (LC-MS), direct-injection mass spectrometry (DIMS), and capillary electrophoresis-mass spectrometry (CE-MS), each of them has its advantages and limitations [81,82].

The GC-MS method is considered one of the most appropriate and inexpensive methods for analyzing metabolites, but this method can only analyze volatile metabolites or those that can volatilize, such as most amino acids, sugar alcohols, aromatic amines, and organic acids [83,84]. The LC-MS method has excellent performance and resolution, simple sample preparation, and the ability to analyze metabolites of different classes, and when using different chromatographic columns, it allows more metabolites to be covered and analyzes polar and non-polar compounds [85,86]. However, this method also has a matrix effect [87], and liquid chromatography introduces additional variability in the metabolic profile of the sample. DIMS and CE-MS methods also have a significant impact on mass spectrometry-based metabolomics diagnostics [88,89,90]. The separation science community considers CE-MS as technically challenging and less reproducible than GC-MS and LC-MS, which, together with the lack of standard operating procedures (SOPs), has led to the limited use of CE-MS [91]. DIMS is attractive as a fast and more reproducible analysis than the “hyphenated” approaches due to the absence of an additional stage of separation of substances by chromatography. DIMS most closely reflects the metabolic profile of the sample, which, however, does not eliminate the negative effects of ion suppression manifested in this approach.

Along with mass spectrometry, NMR spectroscopy has evolved as the most common technique in metabolomics studies. Unlike mass spectrometry, NMR spectroscopy is quantitative and does not require extra steps for sample preparation, such as separation or derivatization. Although the sensitivity of NMR spectroscopy has increased enormously and improvements continue to emerge steadily, this remains a weak point for NMR compared with mass spectrometry that can be considered as a more sensitive and selective platform for metabolomics studies [92].

In addition to equipment, various non-experimental factors in metabolomic research can greatly influence the results. Various physiological factors, such as gender, age, diet, and other external factors, and even changes in the climatic environment, can affect the metabolite composition of the studied objects, which complicates metabolomic data analysis [93,94,95,96,97,98]. In this regard, strict adherence to SOPs and quality control at every stage in the metabolomic study is especially important as it will reduce preanalytical variations and standardize the research process.

Among the limitations of the use of metabolomics based on mass spectrometry in medicine is the problem of measuring the concentration of metabolites in absolute values. The intensity of the mass peak is directly proportional to the level of the metabolite in the measured sample; however, converting the intensity of the peak to absolute values requires the use of chemical standards, which are introduced into the sample in a known amount as a calibrant. Unfortunately, in the case of measuring hundreds of metabolites in metabolomics measurements, this is problematic. An available and compromise solution, in this case, is to compare the intensity of the metabolite peaks in the patient sample with the intensity of the same metabolites in the control group, which allows setting threshold values of the norm and deviations.

Effective identification of metabolites is also a challenge faced by metabolomics [99,100]. The fragmentation patterns of metabolites are quite unpredictable and highly dependent on the characteristics of the equipment. Different metabolites with similar structures often produce the same fragmented ions. There are no chemical standards for confirmation of identifications and quantitative measurement of most metabolites [101,102]. Furthermore, when analyzing metabolites in the metabolomic pathways of live organisms, various analytical methods may show unsatisfactory results [103,104]. Constantly improving analytical hardware and software [105] allows these problems to be solved better. The most significant in terms of personalized medicine seems to be the use of metabolite set enrichment analysis (MSEA) [106]. Such an analysis allows using the power of metabolomics, such as the detection of numerous metabolites simultaneously, while it is tolerant to low reliability in the identification of individual metabolites that takes place in metabolomic measurements. MSEA has the ability to go from statically weak but multiple measurements of metabolites to statistically significant knowledge regarding disease diagnostics or assessment of the organism state, which can be directly accepted by the end-user, such as a doctor. In this case, the need to tinker with the problem of reliable identification of individual metabolites is omitted. According to the authors of the article, this approach is a pillar in the implementation of personalized metabolic analysis, including its implementation in the form of LDT. Thus, the Human Metabolite Database (HMDB; www.hmdb.ca; accessed on 29 April 2021) [107] allows the compilation of metabolite sets associated with 631 diseases and 352 abnormal conditions. By applying MSEA to these sets, the probabilities of diseases and pathological conditions in a patient can be obtained and directly used for medical purposes.

An important and problematic part of metabolomics is the study of the correlation between metabolites and their biological role in diseases. Solving this problem could be the beginning of the transition from biomarkers to mechanisms [69]. For this, many suitable sophisticated bioinformatics tools are currently available, either on the market or free access. Large metabolic databases are available and are endlessly expanding [105].

Thus, metabolomic diagnostics of human health based on mass spectrometry may become a reality, but this requires finding solutions to existing limitations and developing protocols to standardize the research workflow.

## 6. Legal Aspects: LDT Regulation and Logistics

In addition to the technological limitations, the scaling of metabolomic LDTs is also determined by the legal aspects of its regulation and international logistic possibilities. As possible points of growth and scaling of metabolomic LDTs in this context, an overview of three key markets was carried out: the United States, the European Union, and Singapore. These markets were selected based on the presence of the most modern approaches to the regulation of LDTs and the availability of different logistics opportunities.

### 6.1. LDT Regulation in the United States

Despite the long history of the development and formation of potential ways to regulate LDTs from the FDA, at the moment, the laboratories that develop LDTs in the United States are regulated by the CMS under CLIA. All LDTs meet the most stringent CLIA testing category, as high complexity tests.

Along with this, the governments of some states have their requirements for LDTs. For example, New York has its own complex rules for registering LDT [108], while current CLIA regulations only require confirmation of the analytical validity of the LDT, which occurs within 2 years of the initiation of the LDT. The FDA, on the other hand, conducts clinical and analytical validation of IVD tests before their release to the market. It is believed that the approaches to regulating of LDTs by FDA and CMS complement each other.

However, due to the existing uncertainty regarding LDTs, bills are also being created, which are now under consideration. The latest and most relevant bill is the Verifying Accurate Leading-edge IVCT Development Act, the first version of which was released in December 2018 based on the Diagnostic Accuracy and Innovation Act (DAIA) and considering comments from the FDA [109,110]. However, as of on 29 April 2021, there is no effective law, and on 19 August 2020, HHS canceled all previous FDA regulation statements regarding LDTs [33]. Therefore, now, LDTs are regulated with CLIA rules by the CMS.

### 6.2. LDT Regulation in the European Union

IVD medical devices, which, among other things, include LDTs in the European Union until 5 April 2017, were regulated by Directive 98/79/EC of the European Parliament and of the Council on in vitro Diagnostic Medical Devices (IVDMD) [111]. As from 5 April 2017, a new regulation on in vitro diagnostic medical devices was adopted—Regulation (EU) 2017/746 of the European Parliament and of the Council repealing Directive 98/79/EC and Commission Decision 2010/227/EU and establishing a modernized and stronger European Union legal framework to protect public health and patient safety better [112]. 

Among the current changes that will finally come into force in May 2022, several main ones can be distinguished. The examination of biological samples obtained from European citizens is likely to be considered “Distance Selling” (Chapter II, Article 6 of Regulation (EU) 2017/746 of the European Parliament and of the Council of 5 April 2017), which means that such LDTs will require CE IVD labeling regardless of whether the laboratory is located or not located in the European Union. The IVDR will require laboratories to comply with several new standards, including compliance with Annex 1 of the IVDR “General Requirements for Safety and Effectiveness” and the structure of the quality management system and the classification of their LDTs in accordance with Annex VIII of the regulations. In addition, laboratories should, without exception, produce and use LDT “under appropriate quality management systems”, which can be provided by such standards as ISO 13485 and ISO 15189. Based on this, it can be said that the way of regulating LDTs in the European Union is pretty clear, in contrast to the future perspectives of regulation of LDTs in the United States.

### 6.3. LDT Regulation in Singapore

In Singapore, LDTs are regulated by the Health Sciences Authority (HSA) under the Health Products Act (HPA) and its Health Products (Medical Devices) Regulations 2010 [113,114]. In the authors’ opinion, compared to the United States and the European Union, Singapore has the most understandable and effective structure for the classification and regulation of IVD medical devices. On the official website of the Health Sciences Authority of Singapore, it is possible to easily and conveniently determine whether a device is a regulated medical device in 2 steps and, if so, what class it belongs to and what on what rules it is regulated.

If at the first stage the answers to the questions “Is your product manufactured to be used for humans?” and “Is your product intended to provide medical diagnostic information through in vitro diagnostics?” are positive, then the product is defined as a regulated medical device, and it is proposed to proceed to the second stage—the classification of the device.

At the second stage, the number of questions depends on the complexity of the device being classified. In our case, after answering 16 questions, the metabolomic LDT was classified as a class B medical device (“moderate individual risk or low public health risk or both” [115]). This classification is based on GN14 rule 4 (IVD for self-testing) [115]. Metabolomic LDT assumes self-testing using personal kits for self-sampling of capillary blood in the form of a DBS at home and subsequent sending samples to the laboratory. According to GN14 rule 4, metabolomic LDT does not belong to class C because its result is preliminary and does not determine a critical medical condition of a person and requires subsequent confirmation using an appropriate laboratory test [115].

Depending on the device and the scope of diagnostics, as well as some other conditions, there are two ways to register a class B medical device in Singapore: Full registration route and Priority Review Scheme [116,117]. After registering a medical device, the HSA publishes it in the Singapore Medical Device Register (SMDR) [118]. Moreover, for the production of a class B medical device in Singapore, the manufacturer must have a Manufacturer’s license [119].

### 6.4. International Logistics of DBS Samples

As discussed above, one of the main goals of creating a metabolomic LDT is to translate metabolomics into an applied format and make it accessible to a large number of people. One of the determining factors in this context is the convenience of collecting capillary blood samples by people at home and delivery of the DBS samples to the metabolomic laboratory, which has developed and conducts metabolomic research in the LDT format. This prominent advantage to a high degree forms the final appearance of metabolomic LDT, its design, workflow, possible wide applicability, scalability, logistics, and exceptional usefulness for humans. Compatibility with DBS is based on the characteristics of modern mass spectrometers, which allow the measurement of almost all metabolites in a single run for comprehensive analysis using just several microliters of bio sample.

Collecting capillary blood in the form of a DBS using lancets and Whatman^®^ 903 protein saver cards is now widely available, and most self-test kits with this method of biomaterial sampling exist. Subsequent logistics of these samples within the US, EU, and Singapore can also be handled without a problem using most general and specialized postal services.

However, only the international transport of DBS samples can truly make metabolomic LDT based on mass spectrometry available and personalized. “The US Department of Transportation (DOT) and the United States Postal Shipping Service (USPS) consider DBS specimens nonregulated, exempt materials” and “DBS specimens can be shipped by mail or other carriers with no reasonable expectations of occupational exposure to blood or other potentially infectious material” [120]. The basic triple-packaging system must be used to mail DBS samples [120].

Furthermore, DBSs collected by applying a drop of blood onto absorbent material are not subject to UN 3373 Regulations. In addition, DBSs are unregulated materials according to USPS Publication 52, Hazardous, Restricted, and Perishable Mail (section 346.234) [121].

In this regard, it seems possible with the proper existing international packaging standards and the use of the recommended packaging of postal companies to transport DBS samples between different countries, and the absence of strict restrictions in the temperature regime of transportation makes the transportation procedure even easier. Such transportation is possible with the use of companies such as FedEx and UPS between countries in which the transport of dangerous goods is allowed [122,123]. This fact is a great advantage, allowing metabolomic LDT to be implemented only in a few high-tech centers where DBS samples are delivered and processed centrally.

## 7. Conclusions

LDTs have been around for several decades, and traditionally their volume is small for diagnostic applications with a low level of risk to the end-user. Today, there are also quite complex LDTs, which make it possible to diagnose individual health parameters for a wide range of people. Metabolomics, the latest and newest of the omics sciences, has accumulated a large amount of data over the past 10 years that can be used to develop personalized and comprehensive diagnostic metabolomic LDTs that will transfer metabolomics from a scientific field to a practical one and allow many people to study their health condition at the molecular level. Scientists still have to solve many problems in the field of applied metabolomics based on mass spectrometry, but precedents have already been created, and there are many opportunities for introducing metabolomics into our daily life. Compatibility metabolomic LDT with DBS, current legal rules, and postal services rules already today allow mass implementation of metabolomic LDT, such as a worldwide network, where DBS samples are delivered for analysis from different parts of the world to several high-tech centers, thereby delivering this service to everyone.

## Figures and Tables

**Table 1:** Comments on the comparison of metabolomic LDT with clinical blood tests and metabolomic study in science.

Parameter	Metabolomic LDT	Clinical Blood Test	Metabolomics Study
Accessibility	Convenient logistics, availability of test kits, understandable test results.	Many clinical laboratories, understandable results for doctors, and relatively understandable results for people.	Available to scientists. Not publicly available to people.
Legal regulation	Moderate regulation.	Strict regulation.	Generally not regulated.
Samples	Dried Blood Spot (DBS). Small volume, easy collection, the ability to collect capillary blood from a finger at home. Convenience for children.	Venous blood. Large volume. Blood sampling only in the laboratory with the help of a physician. Inconvenience for children.	Generally, blood plasma with a particular anticoagulant (EDTA) is suitable.
Scalability	Good and simple scalability due to the sale of test kits and the possibility of further transportation of DBS samples through the postal service even to another country.	Moderate scalability. Expansion requires opening new laboratories, purchasing expensive equipment, and going through certification and regulatory procedures each time.	Not scalable. Each scientific laboratory is unique.
Test output (result, report)	Understandable to most people. In one analysis, it is possible to analyze many metabolomic pathways, confirm general health status, or identify potential abnormalities at the deep molecular.	Understandable to doctors. To analyze many analytes, a large volume of blood is required, and many separate tests are needed. It is expensive for comprehensive health diagnosis.	The results of scientific research are very complex and are intended for scientists with experience in the same field of science.
Speed (time expenditure)	Fast testing speed due to the automation of all processes. Testing speed may change in the case of sending DBS samples to another country.	High testing speed due to the presence of laboratories in the city. The speed may increase due to the amount of analyzed biomaterial.	Very low. Scientific study can take from several months to several years.
Cost	Moderate. The sample processing rate is relatively low due to their sequential processing by mass spectrometers.	Low for one or more measured parameters. Moderate when measuring a large number of parameters.	Very high. Individual design of each study, highly qualified staff, a large volume of work, long-term implementation.
Educational effect	Any interested citizen can engage in in-depth research of his body using Metabolomic LDT, thereby receiving personalized data and being able to track their change over time.	The purpose and parameters of a clinical blood test are usually prescribed by a doctor and, therefore, as a rule, are not used independently by citizens for self-monitoring of their state of health.	Restricted educational effect for the general public. The results of scientific study are mainly intended for scientists.

## Abbreviations

Laboratory-developed tests (LDTs), in vitro diagnostic devices (IVDs), US Food and Drug Administration (FDA), Clinical Laboratory Improvement Amendments (CLIA), Centers for Medicare and Medicaid Services (CMS), Federal Food, Drug, and Cosmetic Act (FD&C Act), human papillomavirus (HPV), Dried Blood Spot (DBS), nuclear magnetic resonance (NMR), gas chromatography-mass spectrometry (GC-MS), liquid chromatography-mass spectrometry (LC-MS), direct-injection mass spectrometry (DIMS), capillary electrophoresis-mass spectrometry (CE-MS), standard operating procedures (SOPs), metabolite set enrichment analysis (MSEA), the Human Metabolite Database (HMDB), in vitro Diagnostic Medical Devices (IVDMD), Health Products Act (HPA), Singapore Medical Device Register (SMDR), Health Sciences Authority (HSA), US Department of Transportation (DOT), United States Postal Shipping Service (USPS).
